# Attenuation of Strychnine-Induced Epilepsy Employing *Amaranthus viridis* L. Leaves Extract in Experimental Rats

**DOI:** 10.1155/2023/6684781

**Published:** 2023-03-14

**Authors:** Aashish Bharadwaj, Ashwani Sharma, Talever Singh, Devender Pathak, Tarun Virmani, Girish Kumar, Anjali Sharma, Abdulsalam Alhalmi

**Affiliations:** ^1^School of Pharmaceutical Sciences, MVN University, Palwal, Haryana 121105, India; ^2^Rajiv Academy for Pharmacy, Chhatikra, Mathura 281003, India; ^3^Freelancer, Pharmacovigilance Expert, India; ^4^Department of Pharmaceutical Science, College of Pharmacy, Aden University, Aden, Yemen

## Abstract

**Objective:**

Epilepsy is one of the most prevalent neurological illnesses defined by periodic seizures with or without loss of consciousness caused by aberrant neural activity. There are many allopathic medications available for the treatment of epilepsy such as phenytoin (PHY), but the side effects are a major concern. Therefore, the present study involved the evaluation of the pharmacological significance of *Amaranthus viridis* L. extract (EAV) in the management of strychnine (STR)-induced epilepsy.

**Method:**

STR (3.5 mg/kg, i.p.) was injected into male rats 30 minutes after the pre-treatment of a standard drug (PHY: 20 mg/kg) and the two doses of EAV (EAV-200 and EAV-400 mg/kg, p.o.) to the respective groups to cause the convulsions. The anti-convulsant effect of EAV-200 and EAV-400 against STR-induced convulsion in rats was investigated in terms of convulsion onset, duration of convulsions, number of convulsions, and convulsion score. Furthermore, the mitochondrial function and integrity in the brain's prefrontal cortex (PFC) were also estimated.

**Results:**

EAV-400 significantly increased the onset of convulsion from 61.67 ± 3.051 to 119.2 ± 2.738 and reduced the STR-induced duration of convulsions from 144.8 ± 3.582 to 69.17 ± 3.736, number of convulsions from 4.000 ± 0.1592 to 1.533 ± 0.1542, and convulsion score from 5.000 ± 0.3651 to 2.833 ± 0.3073 in rats. EAV-400 significantly attenuated the STR-induced decrease in the mitochondrial function and integrity of the rat PFC. In rats, EAV-400 significantly accelerated the onset of convulsions while decreasing the STR-induced duration, frequency, and score.

**Conclusion:**

Based on investigational findings, EAV-400 could be inferred to be a possible anti-epileptic option for the treatment of epilepsy of this plan in preclinical research.

## 1. Introduction

Epilepsy is a condition in which the balance of neurotransmitters and neuromodulators is disturbed, and it is one of the most often occurring neurological disorders related to unusual, hypersynchronous neuronal activity, characterised by recurrent seizures both with and without unconsciousness [1–3]. In India, it is believed that over 10 million people suffer from epilepsy [4]. As per the World Health Organization (WHO), there are approximately 50 million persons with epilepsy globally, with 80% of them living in developing countries such as India [5].

“Epileptic seizure” is known as a seizure caused by abnormal neural activity as opposed to a non-epileptic event, for example, a psychogenic seizure. The disorder known as “epilepsy” is characterised by recurring and unprovoked seizures. Each of the several epilepsy causes reveals a fundamental brain malfunction [6]. A classification system was first published by the International League against Epilepsy (ILAE) in the year 1960, and the last official update was in the year 1989. The classification is as follows.

### 1.1. Generalised Seizures

This type of seizure affects both sides of the brain [7].

#### 1.1.1. Absence Seizures

Previously, this sort of seizure was referred to as a petit mal seizure. This type of seizures typically ends in 2–15 seconds and might happen only a very less times per day or over 100 times each day. These manifest as dull gazing that might be misinterpreted as daydreaming, somatic automatisms such as twitching of the facial or body muscles, lip biting, tripping, or plucking at clothing. The individual will not remember what occurred during the seizure. It primarily affects youngsters between the ages of 4 and 12.

#### 1.1.2. Generalised Tonic–Clonic Seizure

Grand mal seizure was the old name for this type of seizure. During this seizure, arms and legs stiffen initially; this stage is known as tonicity. This phase is followed by the clonic phase when limbs and heads begin to twitch. Generalised seizures, like other seizures, can vary, with most people having only the tonic–clonic phase. Throughout the seizures, the individual may lessen or stop breathing, chew their tongue or the interior of their mouth, or exhibit incontinence. After the seizure, the individual will most likely be disoriented, will forget what occurred, and may need to sleep and have a headache. Depending on the individual, recovery time might range from minutes to hours.

The events that make up this seizure often start with bilateral myoclonic jerks; then, there is a tonic contraction of the muscles in the extremities as well as the axial trunk, which causes the extremities and neck to extend.

#### 1.1.3. Myoclonic Seizures

The patient's body jerks as a result of these seizures, such as arm/leg twitching. Myoclonic seizures normally do not require first aid, but if it is the first seizure, then there is a need to consult a doctor to find out the cause.

#### 1.1.4. Atonic Seizures

These types of seizures are also referred to as astatic seizures. These seizures cause muscles to relax abruptly and lead to collapse without warning. These seizures cause a portion of the entire body to shuffle. This implies that a person's head may abruptly droop, or may sag or perhaps collapse, falling on the floor.

#### 1.1.5. Partial Seizures

Seizures of this sort are the most prevalent. When only one side of the brain is affected, it occurs. These seizures can cause activity to start in one area of the brain and then move to other, or it may remain in the same location. The symptoms vary according to the part of the brain affected. For instance, the capacity to speak will be impaired if a seizure occurs in the speech centre of the brain. If a seizure begins as a partial seizure and subsequently extends to involve the whole brain, it is called a partial seizure secondary [7].

The procedure of transforming a non-epileptic mind into any capable of causing recurring, spontaneous seizures is known as epileptogenesis. The process is believed to be brought on by an imbalance in inhibitory and excitatory actions inside a neuronal network, which causes it to function exceedingly, hypersynchronously, and oscillatory. If this imbalance persists, it can disrupt other neural circuits as well as normal neuronal processing [8, 9]. Epileptogenic networks are broadly dispersed in generalised epilepsies, involving bilateral thalamocortical regions. Neural circuits in one hemisphere, most frequently limbic or neocortical, are involved in networks for focal epilepsies [8, 10]. Epileptogenic networks are caused by an imbalance between excitation and inhibition, which is not always only an increase in excitation or a decrease in inhibition; an aberrant rise in inhibition can also be pro-epileptogenic in specific circumstances, such as the absence of seizures or limbic epilepsies in the developing brain. The vast majority of generalised epilepsies are assumed to have a hereditary origin. In contrast, structural brain abnormalities were assumed the most prominent feature of localised epilepsies, particularly in drug-resistant epilepsy. However, growing cases of hereditary and de-novo genetic alterations have been discovered in non-lesional focal epilepsy [11].

There are many allopathic medications available for the treatment of epilepsy such as phenytoin (PHY), topiramate, tiagabine, oxcarbazepine, zonisamide, vigabatrin, clobazam, felbamate, lamotrigine, and gabapentin, but as we all know, they all show many side effects as given in Table 1. In contrast to synthetics, which are viewed as being hazardous to use for treatment, herbal products now stand for safety. Based on the worldwide problem and epidemiology of epilepsy, it is reasonable to expect that alternate and supplementary medications for epilepsy management are required.

Natural products are a source of bioactive compounds and are utilised as traditional medical treatments in the treatment of a wide range of disorders, including epilepsy, all over the globe [13]. According to a literature review, the plant *Amaranthus viridis* L. extract (EAV) is recommended for the treatment of several disorders, including diabetes, high cholesterol, pyretic and nociceptive pain, hepatoprotective conditions, and anti-oxidant conditions. Although herbs have been valued for their therapeutic, flavouring, and aromatic properties for millennia, the modern era's synthetic goods temporarily overshadowed their significance. However, the slavish reliance on synthetics has ended, and people are going back to nature in search of stability and safety. For the current study, *A. viridis* L. is selected because its traditional uses show its activity against inflammations, abscesses, gonorrhoea, orchitis, and haemorrhoids; its infusion is used to purify the blood; the pounded root is applied against dysentery and eye infection and also showed its neuroprotective activity in neurological disorders [14] but not in strychnine (STR)-induced epilepsy in rats.

## 2. Materials and Methods

### 2.1. Chemicals and Reagents

PHY was obtained from Sigma Aldrich (St. Louis, MO, USA). All other chemicals and reagents of High-performance liquid chromatography (HPLC) and analytical grade were procured from Merck Pvt. Ltd., New Delhi, and Himedia Laboratories Pvt. Ltd., Mumbai, India. Other necessary chemicals were issued from the chemical store of Rajiv Academy for Pharmacy such as dichloromethane, carboxymethylcellulose (CMC) sodium, and sodium chloride.

### 2.2. Collection of Plant Material and Identification

Fresh leaves of *A. viridis* L. were collected from the Mathura region, Uttar Pradesh, India, in 2022. Leaves were identified and submitted on 31 March 2022 as a specimen in Council of Scientific & Industrial Research (CSIR)-National Institute of Science Communication and Policy Research, New Delhi (authentication number of *A. viridis* L. leaves—NIScPR/RHMD/Consult/2022/4055-56). The identification was made through macroscopic examinations of the sample, an in-depth examination of the literature, and a comparison of the sample with real samples kept in the Raw Material Herbarium and Museum in Delhi (RHMD).

### 2.3. Preparation of Extract of Leaves of *A. viridis*

The plant leaf was collected and dried in the shade. The shade-dried leaves were then roughly pulverised. To get the coarse powder, use a mixer grinder and sieve number 60. The weighted, coarsely powdered ingredients were then used for soxhlet extraction, phytochemical studies, and pharmacological studies after being sealed in an airtight container.

A 1000 ml soxhlet apparatus was filled with 350 g of coarsely crushed leaves, and dichloromethane was then extracted for 72 hours with continuous hot percolation. The solvent was removed after extraction, and the extract was then concentrated at room temperature.

### 2.4. Soxhlet Extraction

The soxhlet extraction method is a constant extraction technique that involves repeatedly cycling the same solvent through the extractor. The steps in this process include solvent extraction and evaporation. The drug is subjected to continuous extraction, whereas the solvent vapours are routed to a condenser, as well as the distillate is then returned. The extractor body of a soxhlet apparatus designed for continuous extraction is coupled to a side tube as well as a syphon tube. Conventional couplings are used to connect the extractor's mouth to a condenser and its lower end to a distillation flask. A thimble made of filter paper or thin muslin cloth or the soxhlet device itself can be used to load the powdered, crude medicament. The inner diameter of the soxhlet apparatus corresponds to the diameter of the thimble. The extraction assembly is finished by installing a distillation flask and a condenser. Before heating, the solvent is initially given a chance to fix the powder. Fresh activated porcelain pieces are placed next to the flask to prevent solvent bumping. The level of fluid in the collector and syphon tube is gradually raised by condensed liquid and vapours that pass through the side tube. A syphon is set up, and the extraction chamber's contents are moved to the flask when the liquid nears the point of return. The cycle of evaporation of the solvent and syphoning back could be repeated as often as is practical to ensure successful extraction. Despite being a continuous extraction process, it is only a series of quick macerations.

### 2.5. Phytochemical Analysis of *A. viridis* Leaves [15]

#### 2.5.1. Tests for Carbohydrate

Separately, a little portion of the extract was mixed in 4 ml of distilled water, and then, it was filtered. The following tests were performed on the filtrate to check for the absence of carbohydrates and glycosides.

#### 2.5.2. Molisch's Test

Two millilitres of concentrated sulphuric acid was then put into the test tube after the filtrate had been treated with a few drops of a 1% alcoholic–naphthol solution. When two liquids come together, there should not be a brown ring since that means there are carbohydrates.

#### 2.5.3. Fehling's Test

After being treated with 1 ml of each of Fehling's solutions A and B, the filtrate was boiled in the water bath. A red precipitate confirmed the presence of the carbohydrate.

#### 2.5.4. Test for Pentose Sugar

Treat test solution with HCL, and then heat and add the phloroglucinol crystal which produce a colored compound with high molar absorptivity which indicate the presence of pentose.

#### 2.5.5. Test for Fixed Oil and Fats

*(1) Spot Test [16]*. The absence of an oil stain on the paper, which indicated the absence of fixed oil, was determined by pressing a little quantity of the extract between two sheets of filter paper.

*(2) Saponification Test [17]*. Add a few drops of 0.5 N alcoholic potassium hydroxide and a drop of phenolphthalein individually to a tiny amount of different extracts and boil in a hot water bath for 1–2 hours. The absence of changes implies the absence of oil and fats.

*(3) Test for Glycerine [18]*. Add 10% sodium hydroxide solution after 5 drops of the sample have been treated with 1% sulphate solution. Glycerine is found in the sample, which is confirmed by the creation of a clear blue solution. The cupric hydroxide produced during the process dissolves in glycerine without precipitating.

### 2.6. Test of Proteins

#### 2.6.1. Biuret Test [19]

The violet colour shows the presence of protein in the extract solution (2 ml) and biuret reagents.

#### 2.6.2. Xanthoprotein Test [20]

A yellow precipitate was created when 5 ml of extract solution and 1 ml of nitric acid were heated. A 40% solution of sodium hydroxide was then added after cooling, and an orange colour resulted.

#### 2.6.3. Test for Steroids [21]

*(1) Salkowski's Test*. A few drops of strong sulphuric acid were added to 1 ml of chloroform solution. The presence of phytosterols results in a brown colour.

*(2) Libermann–Burchard's Test*. Acetic anhydride was diluted with a few drops and added to the extract. After heating, strong sulphuric acid was then poured into the test tube from the side, which caused the upper layer to become green and a brown ring to form at the junction of two layers, confirming the presence of steroids.

#### 2.6.4. Test for Glycosides [22]

*(1) Test A*. A total of 200 mg of the drug was extracted with 5 ml of diluted H_2_SO_4_ by warming on a water bath. After filtering, a 5% solution of sodium hydroxide was used to neutralise the acid that was extracted. To make it alkaline, 0.1 ml of Fehling's solutions A and B was added. The mixture was then cooked in a water bath for 2 minutes. A comparison was made between the quantity of red precipitate produced and that produced in test B.

*(2) Test B*. 5 ml of water was added to drug sample and heated on water bath for 2 minutes after being added to 0.1 ml of Fehling's solutions A and B until it turned alkaline. After boiling, the same amount of water was added. It was heated in a water bath for 2 minutes after being added to 0.1 ml of Fehling's solutions A and B until it turned alkaline. A measurement was made of the amount of red precipitate produced. A comparison was made between the quantity of precipitate produced in tests A and B. Additionally, it shows that glycosides exist.

### 2.7. Tests for Cardiac Glycosides

#### 2.7.1. Baljet Test

Picric acid or sodium picrate was added to the extract. The colour orange was developed, and the presence of glycosides is shown.

#### 2.7.2. Legal's Test

The existence of cardiac glycosides was confirmed by the observation of a blood-red colour in the alcoholic solution of extract, 1 ml pyridine, and 1 ml sodium nitroprusside solution.

#### 2.7.3. Keller–Killiani's Test

A mixture of 2 ml of the extract, 3 ml of glacial acetic acid, and 1 drop of 5% ferric chloride was added. The findings were noted after this solution was carefully applied to the surface of 2 ml of concentrated H_2_SO_4_.

### 2.8. Tests for Anthraquinone Glycosides [23]

#### 2.8.1. Borntrager's Test

In a test tube, the test sample was heated for 5 minutes with 1 cc H_2_SO_4_. It was hot-filtered, then chilled, and shaken with an equivalent amount of dichloromethane/chloroform. Separating the lowest layer of dichloromethane or chloroform and shaking it with 1/2 its volume of diluted ammonia in the ammonical layer, no rose pink to red colour was created.

#### 2.8.2. Modified Borntrager's Test

A total of 200 mg of the substance was cooked in 2 ml of diluted H_2_SO_4_. It was then treated for 5 minutes with 2 ml of 5% aqueous ferric chloride. It was mixed with equal parts chloroform and water. The layer of organic solvent was separated, and an equivalent amount of dilute ammonia was added, resulting in a pinkish-red ammonia layer, which indicates the presence of glycosides.

### 2.9. Test for Flavonoids [22]

#### 2.9.1. Shinoda's Test

Drop by drop, strong HCl was added to the extract solution along with a few magnesium turns. After a few minutes, the colours pink, crimson, and red emerged, revealing the presence of flavonoids. After adding a small amount of leftover lead acetate solution, the colour changed.

### 2.10. Test for Fats and Oils

#### 2.10.1. Solubility Test

(i) A few ml of chloroform was added to 2–3 ml of the extract, and solubility was observed.

(ii) A few ml of 90% methanol was added to 2–3 ml of the extract, and solubility was checked.

### 2.11. Test for Tannins and Phenolic Compounds [21]

#### 2.11.1. Ferric Chloride Test

When ferric chloride solution was added to the test solution, a green colour developed, indicating the presence of condensed tannins.

#### 2.11.2. Phenazone Test

A total of 0.5 g of sodium phosphate was added to the test solution, warmed, and filtered. When 2% phenazone solution was added to the filtrate, a huge precipitate was produced, which was frequently coloured, suggesting that tannins are present.

#### 2.11.3. Test for Alkaloids

Separately, the extract was evaporated. Dilute HCL was added to the residue, which was thoroughly shaken and filtered. The following experiments were carried out.

#### 2.11.4. Dragendroff's Reagents

A few drops of Dragendroff's reagents were applied to 2–3 ml of filtrate, and a precipitate was seen. It denotes the existence of an alkaloid.

#### 2.11.5. Mayer's Test

A few drops of Mayer's reagents were applied to 2–3 ml of filtrate, and a precipitate was seen. It shows that alkaloids are present.

### 2.12. Test for Amino Acids [21]

#### 2.12.1. Ninhydrin Test

3 ml of the test solution was treated in a boiling water bath for 10 minutes with 3 drops of 5% ninhydrin solution. Dark blue colour appearance confirms the presence of amino acids.

#### 2.12.2. Experimental Animals and Their Housing

Adult male Albino Wistar rats (180–220 g) of 10–12 weeks were received and used for the study from Animal House, Rajiv Academy for Pharmacy, Mathura. Under regular conditions (25 ± 2°C temperature, 45–55% humid environment, and a 12 h light–12 h dark cycle), the animals were divided into groups and kept in poly-acrylic houses (cages) lined with husks. The animals were given unrestricted access to their usual pellet food and unrestricted water. Food was withheld from the animals for 16–18 hours before the experiments, but they were permitted to drink as much as they wanted. The animal ethical approval was received from Rajiv Academy for Pharmacy, Mathura under the Institutional Animal Ethical Committee (IAEC) of Rajiv Academy in the meeting held on 08 April 2022, and 30 male Albino Wistar rats have been sanctioned under this proposal for a duration of the next 1.5 months with registration no. is 882/PO/Re/S05/CPCSEA.

#### 2.12.3. Experimental Design

The entire experimental regimen was planned to last 14 days. To summarise, all animals were placed into five groups of six, namely control, STR, STR + EAV-200, STR + EAV-400, and STR + PHY. All animals in the STR + EAV-200, STR + EAV-400, and STR + PHY groups received EAV (200 mg/kg, p.o.) [24], EAV (400 mg/kg, p.o.) [24], and PHY (20 mg/kg, i.p.) [25] for 14 days. Except for the control group rats, all other animals were given STR (3.5 mg/kg, i.p.) [26] on the 14th day of the experiment following 30 minutes of drug pre-treatment. For the duration of the experiment, all control group rats received once daily CMC (0.5% w/v) as a vehicle. In the STR, EAV-200, EAV-400, and PHY groups, death rates were 70%, 20%, 30%, and 20%, respectively. All behavioural characteristics were recorded, and the animals were killed via cervical derangement. The cortical area of the rat brain was separated and promptly kept at –80°C for biochemical analysis [27, 28]. For repeatability, all biochemical studies were done twice.

### 2.13. Grouping of Animals

Animal grouping was done in the following manner:

Group 1: normal control: normal saline 0.9% (10 ml/kg) orally for 14 days.

Group 2: disease control: STR (3.5 mg/kg i.p.).

Group 3: standard: PHY (20 mg/kg, p.o.) (STR + PHY 20 mg/kg).

Group 4: extract of *A. viridis* L. 200 mg/kg, p.o. (STR + EAV-200 mg/kg).

Group 5: extract of *A. viridis* L. 400 mg/kg, p.o. (STR + EAV-400 mg/kg).

### 2.14. STR-Induced Convulsion

STR (3.5 mg/kg, i.p.) was injected into rats 30 minutes after the drug administration [26]. The onset, duration, and number of convulsions were observed and recorded. The convulsion signs were observed, and the severity was graded as per the following scale; moreover, the percentage of mortality rate and protection at 10 minutes was recorded.

### 2.15. Effect of EAV-200 and EAV-400 on STR-Induced Brain Mitochondrial Dysfunction

#### 2.15.1. Isolation of Mitochondria from Rat PFC

To separate mitochondria from rat prefrontal cortex (PFC), the conventional procedure of Pedersen et al. (1978) [29] was utilised. The mitochondrial protein concentration in tissue fractions was determined using Lowry et al. [30] standard technique (1951).

#### 2.15.2. Estimation of Mitochondrial Function in Rat PFC

The (3-(4, 5-dimethylthiazol-2-yl)-2,5-diphenyltetrazolium bromide [MTT]) reduction test was used to determine mitochondrial activity in tissue fractions by measuring the amount of formazan produced at 595 nm using a spectrophotometric technique [31]. The results were represented in milligrammes of formazan produced each minute per milligramme of protein.

#### 2.15.3. Evaluation of MMP in Rat PFC

The mitochondrial membrane potential (MMP) was evaluated by measuring the amount of rhodamine dye taken up by mitochondria in a spectrofluorometer (Hitachi, F-2500) at 535 ± 10 nm excitation and 580 ± 10 nm emission [32]. The results were reported in terms of fluorescence intensity per milligramme of protein.

### 2.16. Histological Analysis of Brain

Samples provided were fixed in 10% neutral buffered formalin. Fixed tissues were dehydrated in graded series of 30%, 50%, 70%, 90%, and 100% alcohol to remove water and crystals of picric acid present in Bouins' solution used as fixative. Fixed specimens were dehydrated. Tissues were cleared in xylene and infiltrated with paraffin wax, and sections of uniform thickness (4–6 *μ*) were made by using a microtome. Casting or blocking specimens are embedded in paraffin using embedding rings and orienting tissue to the area of interest. Blocks were placed at 4°C for 15 minutes to solidify. 5 *μ*m sections were cut using a spencer-type rotary microtome. Cut sections were placed in a 45°C water bath and put on sialinated slides. Slides were allowed to dry in a 37°C oven overnight before staining takes place. Hematoxylin and eosin (H&E) dyes were used to stain the sections that were further visualized in a microscope equipped with an Amscope MU1000 camera [33].

## 3. Results

### 3.1. Phytochemical Analysis

The results of the phytochemical analysis are depicted in Table 2.

### 3.2. Mortality Rate and Percent Protection

The effect of EAV-200, EAV-400, and PHY on the mortality rate and percentage protection is shown in Table 3.

From the above table, it can be observed that the mortality rate decreased after the administration of STR + EAV-200 and STR + EAV-400, and the protection increased.

The highest protection was provided by PHY, which is the standard drug followed by EAV-200 and 400 mg/kg.

### 3.3. EAV-200 and EAV-400 mg/kg Attenuates STR-Induced Convulsions in Rats

#### 3.3.1. Onset of Convulsion

**Figure 1:**
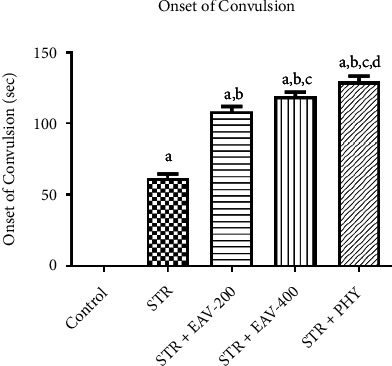
Graph showing the impact of EAV-200, EAV-400, and PHY on strychnine-induced convulsion onset. All values are mean ± SEM (*n* = 6). ^a^*p* < 0.001 compared to control, ^b^*p* < 0.001 compared to STR, ^c^*p* < 0.001 compared to STR + EAV-200, ^c^*p* < 0.05 STR + EAV-400 compared to STR + EAV-200, ^d^*p* < 0.05 STR + PHY compared to STR + EAV-400 (one-way ANOVA followed by Student–Newman–Keuls *post hoc* test).

Rats showed convulsion after administration of PHY.  Figure 1 illustrates the impact of STR + EAV-200, EAV-400, and PHY on the onset of convulsion in STR-challenged rats. The results of the statistical analysis showed in Table 4, which shows an onset of convulsion [*F*(4, 25) = 312, *p* < 0.001]. EAV-400 in the onset of convulsion significantly showed a difference compared to control, STR, and STR + PHY, which states that EAV-400 has the potential to attenuate the convulsion onset.

#### 3.3.2. Duration of Convulsion

**Figure 2:**
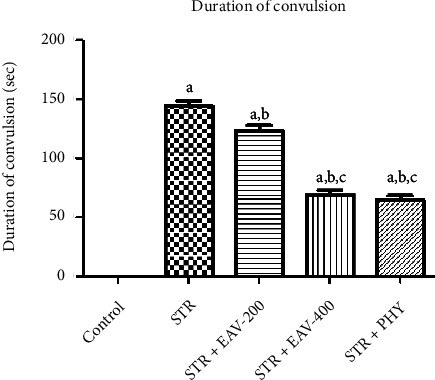
Graph showing the impact of EAV-200, EAV-400, and PHY on the duration of convulsion. All values are mean ± SEM (*n* = 6). ^a^*p* < 0.001 compared to control, ^b^*p* < 0.001 compared to STR, ^c^*p* < 0.001 compared to STR + EAV-200, ^c^*p* < 0.05 STR + EAV-400 compared to STR + EAV-200.

Figure 2 illustrates the impact of STR+EAV-200, EAV-400, and PHY on the duration of convulsions in STR-challenged rats. The results of the statistical analysis showed that the duration of the convulsion has a significant difference [*F*(4, 25) = 246.5, *p* < 0.001]. EAV-400 in the duration of convulsion showed a significant difference when compared to control, STR, and STR + EAV-200, which states that EAV-400 has the potential to attenuate the duration of convulsion. The figure is showing the relationship between STR, EAV-200, EAV-400, and PHY in the duration of convulsions. The duration of convulsion was decreased by the administration of EAV-400 in comparison to STR. EAV-400 has provided a much better effect in comparison to EAV-200 and decreased the duration of convulsions.

#### 3.3.3. Number of Convulsions

**Figure 3:**
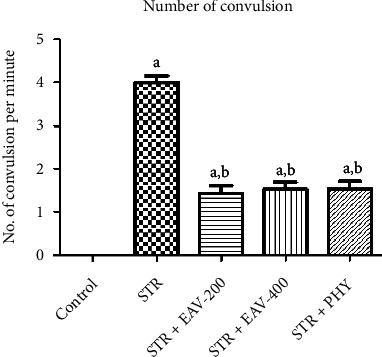
Graph showing the impact of EAV-200, EAV-400, and PHY on the number of convulsions per minute. All values are mean ± SEM (*n* = 6). ^a^*P* < 0.001 and ^b^*P* < 0.001 compared to control and STR, respectively (one-way ANOVA followed by Student–Newman–Keuls *post hoc* test).

Figure 3 illustrates the impact of STR+EAV-200, EAV-400, and PHY in the number of convulsions in STR-challenged rats. The results of the statistical analysis showed in Table 5, and the number of convulsions has a significant difference [*F*(4, 25) = 91.95, *p* < 0.001]. EAV-400 in several convulsion significantly showed a difference compared to control, STR, and STR + EAV-200 but not showed a significant difference compared to STR + PHY, which states that EAV-400 have the potential to attenuate the duration of convulsion. The number of convulsions per minute was also recorded, which is shown in Figure 3. As it is evident from the figure, the rats treated with STR suffered an average of 4 convulsions per minute, which was decreased to an average of 1–2 convulsions per minute with the help of EAV-200, EAV-400, and PHY.

#### 3.3.4. Convulsion Score

**Figure 4:**
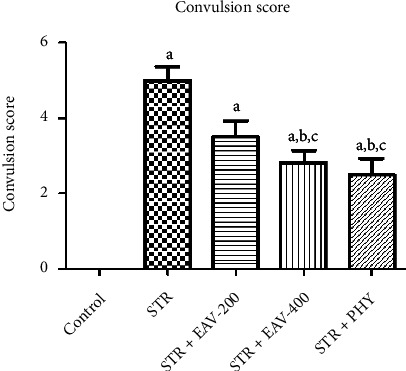
Graph showing the impact of EAV-200, EAV-400, and PHY on convulsion scores. All values are mean ± SEM (*n* = 6). ^a^*P* < 0.001, ^b^*P* < 0.001 compared to control and STR, respectively (one-way ANOVA followed by Student–Newman–Keuls *post hoc* test), ^c^*P* < 0.001 compared to STR + EAV-200.

Figure 4 illustrates the impact of STR + EAV-200, EAV-400, and PHY on convulsions score in STR-challenged rats. The results of the statistical analysis are shown in Table 6, in that the number of convulsions has a significant difference [*F*(4, 25) = 38.06, *p* < 0.001]. EAV-400 in convulsion score significantly showed a difference compared to control, STR, and STR + EAV-200 but not showed a significant difference compared to STR + PHY which states that EAV-400 have the potential to attenuate the severity of convulsion.

As discussed earlier, scores were given to the rats based on the severity of the convulsions they are getting. In Figure 4, it is demonstrated that the convulsion scores were highest in the case of STR which gradually decreased after the administration of EAV-400. PHY has demonstrated the least convulsion score.

### 3.4. EAV-400 Attenuates STR-Induced Decrease in Mitochondrial Function and Integrity in Rat PFC

**Figure 5:**
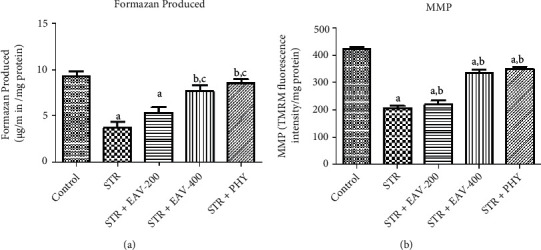
Graph showing changes in the mitochondrial function in terms of the level of formazan produced in MTT assay and changes in the integrity in terms of the fluorescence intensity of TMRM. All values are mean ± SEM (*n* = 6). ^a^*P* < 0.001, ^b^*P* < 0.001 compared to control and STR, respectively (one-way ANOVA followed by Student–Newman–Keuls *post hoc* test), ^c^*P* < 0.001 compared to STR + EAV-200.

The effect of EAV-400 on the STR-induced changes in the mitochondrial function in terms of the level of formazan produced in MTT assay (A) and integrity in terms of the fluorescence intensity of tetramethylrhodamine, methyl ester (TMRM) (B) in rat PFC is illustrated in Figure 5, and results are given in Table 7. The statistical analysis is shown in the table, which revealed that there were significant differences in mitochondrial function and integrity in rat PFC ([*F*(4, 25) = 16.21, *P* < 0.001] and [*F*(4, 25) = 77.92, *P* < 0.001], respectively). Post hoc analysis showed that EAV-400 and PHY significantly increased the STR-induced decrease in mitochondrial function and integrity in rat PFC. Moreover, there were no significant differences in mitochondrial function and integrity of rats PFC among STR + EAV-400 and STR + PHY groups.

MTT is reduced to formazan in the mitochondria, an increase in the level of formazan increases the mitochondrial mass thereby increasing metabolic feasibility. As it is evident from the graph, the amount of formazan produced was highest in the case of PHY and lowest in the case of STR. Whereas, EAV-400 helped to produce more formazan in comparison to STR and EAV-200. Further, PHY increased the production of formazan in the highest amount.

TMRM is localized in mitochondria and used to detect mitochondrial membrane depolarization. MMP is very high in the case of the normal control group, which is decreased by the STR treatment. EAV-400 has gradually increased the MMP and is highest in the case of PHY.

### 3.5. Histological Analysis of the Brain with H&E Staining

**Figure 6:**
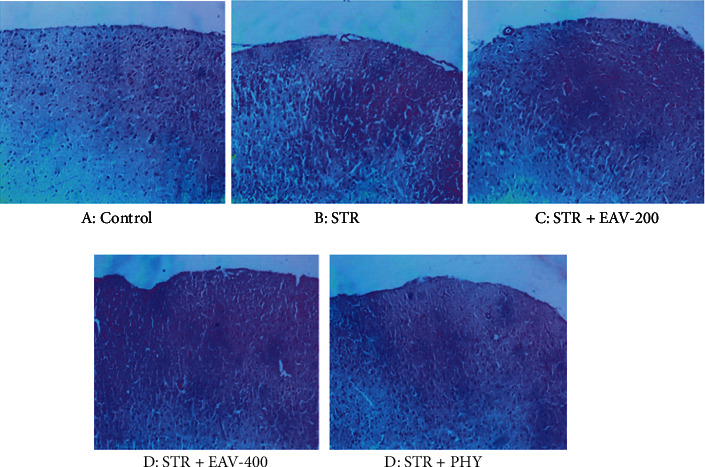
Shows the differences anatomically in STR-induced duration of convulsion, STR + EAV-200, STR + EAV-400, control, and PHY.

Figure 6 shows the differences anatomically in STR-induced duration of convulsion, STR + EAV-200, STR + EAV-400, control, and PHY.

## 4. Discussion

The current work indicates for the first time that EAV-400 is neuroprotective against STR-induced convulsions in experimental rats. Furthermore, the level of neuroprotection provided by EAV-400 was comparable in the experimental rats. EAV-400 is found to reduce the STR-induced mitochondrial dysfunction in rats' PFC. As a result, EAV might be regarded as an alternate approach to epilepsy management.

STR caused convulsions with a 70% fatality rate in rats in the current investigation, which was similar to previous studies [34]. EAV-400 considerably delayed the start of STR-induced convulsions in the rats. Furthermore, EAV-400 considerably decreased the length, frequency, and severity (score) of STR-induced convulsions in rats. Furthermore, compared to the previous trial, the anti-convulsant action of the EAV was found statistically equivalent to that of the conventional medication PHY [35].

These observations clearly demarcate the fact that EAV possesses glycine receptor agonist activity in the brain.

It is widely established that mitochondria play a vital role in the development of neurons at the subcellular level [36]. As a result, the current investigation of the mitochondrial basis of EAV-400's anti-convulsant action against STR-induced convulsion in experimental rats. EAV-400 significantly reduced STR-induced decrements in mitochondrial function and integrity in the rat PFC in the current investigation.

## 5. Conclusion

Finally, EAV-400 demonstrated anti-convulsant efficacy in a STR-induced animal model of epilepsy. Furthermore, EAV-400 reduced STR-induced mitochondrial damage in rat PFC. As a result, EAV might be regarded as an additional preventative strategy in the treatment of epilepsy. Furthermore, including EAV in the dietary diet can help lower-income people avoid malnutrition-induced epilepsy. Based on investigational findings, EAV-400 could be inferred to be a possible anti-epileptic option for the treatment of epilepsy of this plan in preclinical research.

## Figures and Tables

**Table 1:** Side effects of various anti-epileptic drugs.

Drug	The main side effects	Important but very rare side effects	Reference
Topiramate	Cognitive difficulties, tremors, dizziness, ataxia, headache, fatigue, gastrointestinal upset, and renal calculi		Dichter and Brodie [12]
Tiagabine	Confusion, dizziness, gastrointestinal upset, anorexia, and fatigue		
Oxcarbazepine	Dizziness, diplopia, ataxia, headache, weakness, rash, and hyponatremia		
Zonisamide	Somnolence, headache, dizziness, ataxia, and renal calculi		
Vigabatrin	Behavioural changes, depression, sedation, fatigue, weight gain, and gastrointestinal upset	Psychosis	
Clobazam	Sedation, dizziness, irritability, depression, and disinhibition		
Felbamate	Irritability, insomnia, anorexia, nausea, and headache	Aplastic anaemia and hepatic failure	
Lamotrigine	Rash, dizziness, tremor, ataxia, diplopia, headache, and gastrointestinal upset	Stevens–Johnson syndrome	
Gabapentin	Somnolence, fatigue, ataxia, dizziness, and gastrointestinal upset		
Phenytoin	Phenytoin encephalopathy, psychosis, locomotor dysfunction, hyperkinesia, megaloblastic anaemia, decreased bone mineral content, Stevens–Johnson syndrome, toxic epidermal necrolysis, immunoglobulin. A deficiency, gingival hyperplasia, dress syndrome (drug reaction accompanied by eosinophilia and systemic symptoms), cardiovascular collapse, hypotension, arrhythmias, hydantoin syndrome in newborns, and purple glove syndrome		

**Table 2:** Outcomes of phytochemical analysis.

S. no.	Constituents	Dichloromethane extract of leaves of *A. viridis* L.
1.	Carbohydrates	Positive
2.	Fixed oils and fats	Negative
3.	Protein and amino acid	Positive
4.	Saponins	Positive
5.	Steroids	Positive
6.	Alkaloids	Positive
7.	Glycosides	Positive
8.	Flavonoids	Positive
9.	Tannins	Positive
10.	Gum and mucilage	Negative
11.	Triterpenoids	Positive
12.	Phenolic compounds	Positive

**Table 3:** Effect of EAV-200, EAV-400, and PHY on mortality rate and percentage protection.

Groups	Number of animals		Mortality rate (%) (10 minutes)	Protection (%) (10 minutes)
	Dead	Alive		
Control	0	6	0	100
STR	4	2	67	33
STR + EAV-200	3	3	50	50
STR + EAV-400	2	4	34	66
STR + PHY	1	5	17	83

**Table 4:** Impact of EAV-200, EAV-400, and PHY on convulsion onset.

Groups	The onset of convulsion (seconds)
Control	0 ± 0
STR	61.67 ± 3.051^a^
STR + EAV-200	108.5 ± 3.748^a,b^
STR + EAV-400	119.2 ± 2.738^a,b,c^
STR + PHY	137.2 ± 4.542^a,b,c,d^

All values are mean ± SEM (*N* = 6).

^a^ *p* < 0.001 compared to control.

^b^ *p* < 0.001 compared to STR.

^c^ *p* < 0.001 compared to STR + EAV-200.

^c^ *p* < 0.05 STR + EAV-400 compared to STR + EAV-200.

^d^ *p* < 0.05 STR + PHY compared to STR + EAV-400.

One-way ANOVA followed by Student–Newman–Keuls *post hoc* test.

**Table 5:** Impact of EAV-200, EAV-400, and PHY on number of convulsions per minute.

Groups	Number of convulsion per minute
Control	0 ± 0
STR	4.000 ± 0.1592^a^
STR + EAV-200	2.917 ± 0.2286^a,b^
STR + EAV-400	1.533 ± 0.1542^a,b^
STR + PHY	1.550 ± 0.1565^a,b^

All values are mean ± SEM (*N* = 6).

^a^ *p* < 0.001 compared to control.

^b^ *p* < 0.001 compared to STR.

One-way ANOVA followed by Student-Newman–Keuls *post hoc* test.

**Table 6:** Impact of EAV-200, EAV-400, and PHY on convulsion scores.

Groups	Convulsion score
Control	0 ± 0
STR	5.000 ± 0.3651^a^
STR + EAV-200	3.500 ± 0.4282^a^
STR + EAV-400	2.833 ± 0.3073^a,b,c^
STR + PHY	2.500 ± 0.4282^a,b,c^

All values are mean ± SEM (*N* = 6).

^a^ *p* < 0.001 compared to control.

^b^ *p* < 0.001 compared to STR.

^c^ *p* < 0.001 compared to STR + EAV-200.

One-way ANOVA followed by Student–Newman–Keuls *post hoc* test.

**Table 7:** Changes in the level of formazan produced in the MTT assay.

Groups	MMP	Formazan produced
Control	422.5 ± 9.106	9.333 ± 0.4944
STR	207.7 ± 8.876^a^	3.667 ± 0.6667^a^
STR + EAV-200	219.8 ± 13.73^a,b^	5.333 ± 0.6146^a^
STR + EAV-400	337.7 ± 10.30^a,b^	7.667 ± 0.6667^b,c^
STR + PHY	349.8 ± 9.062^a,b^	8.500 ± 0.4282^b,c^

All values are mean ± SEM (*N* = 6).

^a^ *p* < 0.001 compared to control.

^b^ *p* < 0.001 compared to STR.

^c^ *p* < 0.001 compared to STR + EAV-200.

One-way ANOVA followed by Student–Newman–Keuls *post hoc* test.

## Data Availability

The supporting literature, data, and other necessary information used to support the findings of this study are available from the corresponding author upon request.
